# Design and Initial Evaluation of a Novel Oral Hygiene Technology for a Special Needs Population: A New Way to Clean

**DOI:** 10.3390/dj11090224

**Published:** 2023-09-20

**Authors:** Maxine Strickland, Steven Mills, Bhargavi Dasari, Kenneth Markowitz, Carla Cugini

**Affiliations:** 1 Department of Diagnostic Sciences, Rutgers School of Dental Medicine, 110 Bergen Street, Newark, NJ 07101, USA; mac.mills513@gmauil.com (S.M.); bhargavibds@gmail.com (B.D.); 2 Department of Oral Biology, Rutgers School of Dental Medicine, 110 Bergen Street, Newark, NJ 07101, USA; markowkj@sdm.rutgers.edu (K.M.); cc1337@sdm.rutgers.edu (C.C.)

**Keywords:** powered toothbrush, oral hygiene, microbiological methods

## Abstract

9.4 million People have swallowing problems in the US. In special needs populations, routine oral hygiene procedures such as tooth brushing can result in aspiration of microbial laden fluids leading to a significant systemic challenge. Aspiration may lead to pneumonia in susceptible populations. These circumstances indicate the need for innovative approaches to oral hygiene for special needs, convalescent, the elderly populations, and young children learning to brush who can ingest excess fluoride which causes mottled enamel. Methods include describing some of the design considerations of the new prototype fabrication and microbiological evaluation of this new device, as well a comparison study of the versions 2 and 3 of the oral care device. Results concluded that version 3.0 regarding patient ease of use was better in comparison to version 2, which was the major difference, and 90% in both groups said they would recommend the new toothbrush. In the microbiological evaluation no growth was seen on any plates containing samples from either the experimental or the control after 48 h of incubation.

## 1. Introduction

Oral hygiene is an important determinant of oral and systemic health, especially for the critically ill, geriatric, disabled and pediatric populations. Poor oral hygiene has been causally linked to many common dental diseases such as caries and periodontal disease. 

Poor oral hygiene leading to plaque accumulation causes an imbalance between microorganisms and the host immune system. This is accompanied by changes in the composition of the bacterial environment and composition of the plaque. This process of dysbiosis frequently allows disease-associated organisms to increase their proportions in the plaque community. Poor oral hygiene in special needs populations is commonly encountered. This impacts the oral microbiome in these individuals. In a study where 61 children with autism spectrum disorder (ASD) age 6–16 and poor oral hygiene were compared with children without autism, the investigators found that a lower diversity of bacteria existed in the ASD patients when compared to healthy subjects. In persons with ASD a positive relationship was observed between plaque levels and certain bacterial taxa such as *Streptococcus*, *Actinomyces* and *Capnocytophagia*. Similar changes favoring less diversity were observed in the gut flora of individuals with ASD. ASD subjects also had higher decayed, missing, and filled teeth [1,2]. 

In individuals with poor hygiene dysbiosis, may also be influenced by the immune system and genetics. When the microbial environment favors an inflammophilic species and the vascular events underlying inflammation, we see the delivery of circulating nutrients to the subgingival environment and increase in bacterial growth. This is an important step in the development of gingivitis and other forms of periodontal disease. Keystone pathogens can avoid detection and destruction by the host response while benefiting from increased nutrient delivery. An example of a keystone pathogen that thrives in patients with poor oral hygiene and linked to systemic illness is Porphyromonas gingivalis. This organism shuts down the body’s immune response causing chemokine paralysis. Neutrophils will enter the connective tissue but are not initially directed to the site of infection and their killing mechanism is inhibited leading to excessive inflammation [3,4,5]. A study conducted in young Japanese children with special needs observed periodontal pathogens in many subjects [6].

In addition to the dental health, and the impact of poor oral hygiene, there are wide ranging systemic consequences. Of relevance to special needs populations is the risks and sequela of plaque, bacterial aspiration, and pneumonia can occur after aspiration of fluids containing microorganisms from the oral cavity. This is a common problem in the elderly, in nursing homes and in the disabled patient. A review of randomized control trials of aging population revealed that oral hygiene had a positive impact on pneumonia and respiratory infections which was reduced from 19–11.0% [7]. This presents a challenge to patients and caregivers. Oral hygiene practices are critical in preventing dysbiosis and alternatively, persons with poor motor function may aspirate bacteria-laden oral fluids during oral hygiene practices. 

Results suggest that the need for assistance with dental hygiene interventions might be one mechanism to minimize harmful microbial changes in those that are neurodivergent. Prevention involves meticulous professional cleanings and oral care to remove plaque and calculus deposits, performed by the patient or care giver. This prevents the anerobic environment that is suited to many keystone pathogens in the plaque environment. Dental professionals can minimize the risk of local and systemic sequela by optimizing oral hygiene performance. A systematic review and meta-analysis revealed that when dental personnel provided intense patient’s hygiene interventions, the risk of hospital-acquired pneumonia (HAP) and HAP-related mortality was less than half the risk when compared to the patients usual oral care (RR = 0.43, 95% CI = 0.25–0.76, *p* = 0.003) and the numbers needed to treat (NNT 8.6–11.3) indicating that this intervention prevents one in ten deaths [8].

Data obtained from the general population supports the use of powered tooth brushing as a means of plaque removal when compared to a manual toothbrush, in a 2014 Cochrane systematic review. It was concluded that there is moderate quality evidence that powered toothbrushes provide a statistically significant benefit compared with manual toothbrushes regarding the reduction of plaque in both the short term (standardized mean difference (SMD) −0.50 (95% confidence interval (CI) −0.70 to −0.31); 40 trials, *n* = 2871) and long-term (SMD −0.47 (95% CI −0.82 to −0.11; 14 trials, *n* = 978) [9]. The use of powered toothbrushes may increase the risk of aspiration due to the vigorous fluid movement generated during use. This was demonstrated in a pilot study after consent and IRB approvals the study was conducted on 11 young (median age 16) disabled individuals in a residential facility. The subjects assisted by their caregiver conducted tooth brushing with a power brush while being evaluated using fiberoptic endoscopy (FEES) it was discovered that patients were aspirating during their toothbrushing. Aspiration was also assessed during manual toothbrush use. Oral hygiene scores were assessed at the beginning of the study. Subjects found to aspirate using the power toothbrush were switched to a manual toothbrush and again assessed for aspiration using FEES. The subjects then used either the power or manual brush for 2 months following which their oral hygiene was reassessed. Overall, the study revealed that 5 subjects (45.5%) aspirated during power toothbrush use. Only one subject 0.9% aspirated during manual toothbrush use. Subjects using the power brush experienced greater improvements in plaque levels and bleeding scores than the subjects using the manual brush for 2 months. [10] These findings, indicate that power tooth brushing can be valuable in assisting oral hygiene in individuals with special needs if the risk of aspiration could be mitigated.

The new technology, the MaxVac powered toothbrush is aimed to prevent aspiration, respiratory diseases, and various comorbidities by reducing bacterial contamination from excess fluids in the oral and pulmonary cavity that can lead to pneumonia. Not only would this technology be beneficial to the elderly, the disabled and the unconscious but it would also prove to be useful to the young children just learning to brush to prevent ingesting excess fluoride when brushing. Such ingestion can cause what’s known as mottled enamel.

One important goal in designing MaxVac is to provide a changeable toothbrush head since all toothbrush heads are subject to wear and are usually used for a period of about 3 months after which they are discarded as recommended by the American Dental Association. Discarding the toothbrush head is a part of good oral hygiene since a new toothbrush head performs better as demonstrated in a recently published study with a follow-up of 1 year, all participants performed a similar basic home-based oral hygiene regimen including specific toothbrush use instructions. Toothbrushes were turned in every 3-months, and the degree of wear was scored. Patients whose toothbrushes had more wear had more plaque remaining [11].

Our initial evaluation of the MaxVac powered toothbrush had two purposes: (1) to demonstrate the ease of which to produce replacement heads for the new toothbrush system, and (2) evaluate a simple but effective method for cleaning the internal portion of the toothbrush system between uses with a commonly uses commercial mouthwash. This research aims to study the cleaning ability of Listerine for a novel powered toothbrush with suction. In addition, a survey was done with individuals who used the toothbrush, in order to assess the acceptability of the device. 

Students at the Rutgers School of Dental Medicine participated in running the experiments set up by the principal investigator. The learning objective underlying the student’s participation was to collect survey data and understand analysis as it pertains to clinical relevancy in evidence-based practice for this population as patients used the toothbrush. Clinical relevancy included the sterilization protocols which reduced the bacterial load inside the devices system to undetectable amounts of bacteria that could not cause illness to users.

## 2. Methods

### Prototype Design Manufacturing

The first step in developing an assistive toothbrush technology embodied by the MaxVac powered toothbrush was prototyping an automatic device with evacuation capabilities. A proof of concept was demonstrated with a snap on vacuum to an existing toothbrush as version one and the initial patent. The next two prototypes were innovative prototypes that centered on being an efficacious handheld all in one electric toothbrushes with a hollow brush head and built-in vacuum and a collection reservoir. Interestingly, this gap vacuum while brushing has not been addressed outside of the dental clinic in-patient services. Products that employ suction are bulky, with either brush or vacuum not both and have exterior collection bags. These devices have had poor market penetrance.

**Figure 1:**
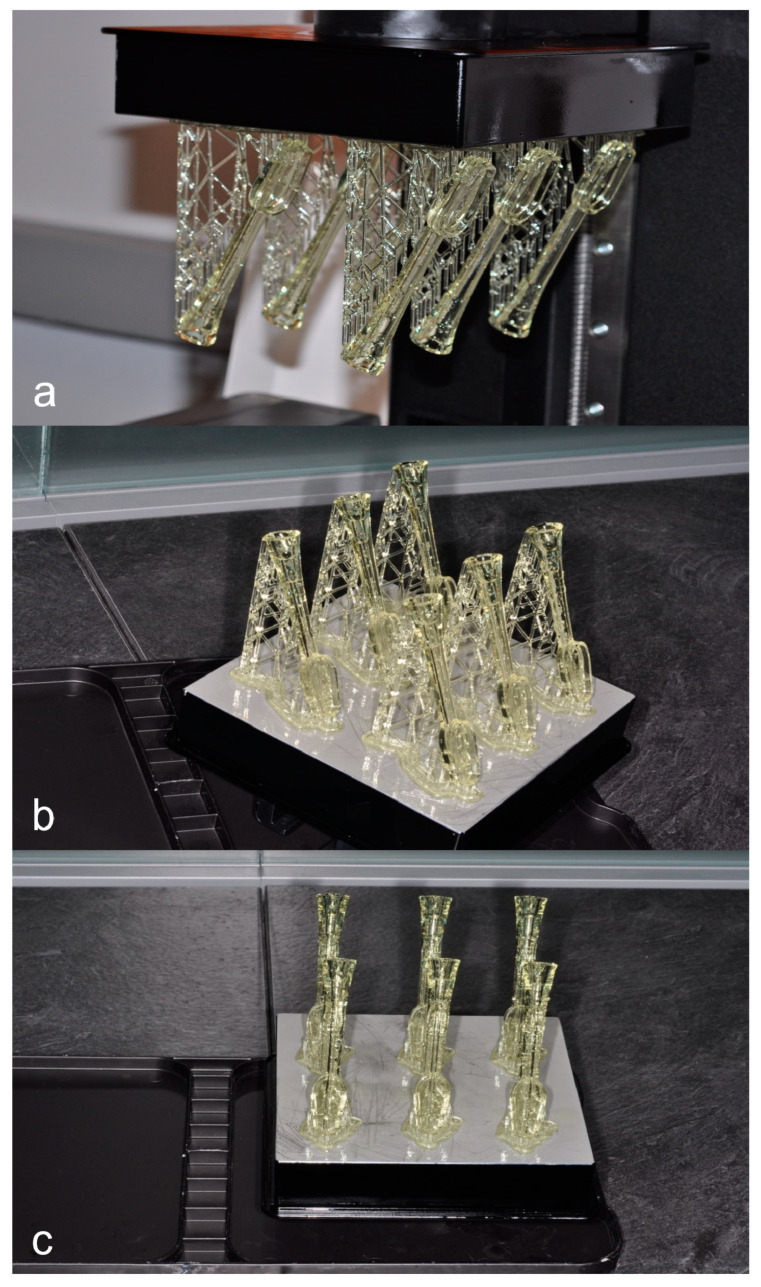
(**a**). 3D printed toothbrush heads. (**b**). 3D printed toothbrush heads. (**c**). 3D printed toothbrush heads successfully done.

Our design features a novel all-in-one device including an electric vacuum to allow for continuous suction even when manual strength and dexterity are a concern for users. 3D printing technology offered a practical and flexible means to rapidly design, fabricate, and modify prototypes allowing for rapid design evolution. The 3D printer was used at the Rutgers digital center (Figure 1). After loading the resin, it would take several hours to complete the printing process. Afterwards we performed several steps of water then alcohol washings to remove unpolymerized resin.

**Figure 2:**
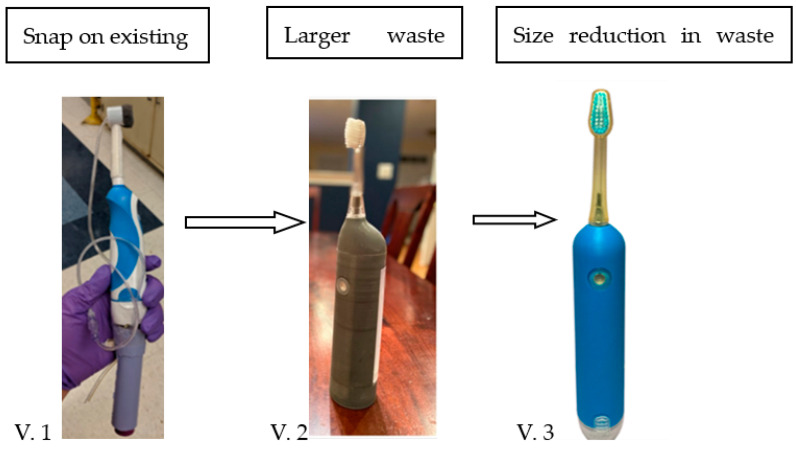
Evolution of the MaxVac saliva collection capacity.

Prototypes (Figure 1) toothbrush heads were fabricated at the digital center at the Rutgers School of Dental Medicine. Features are a hollow toothbrush head, and a compact head designed to be comfortably maneuvered during toothbrushing. The waste reservoir is a separate compartment to collect oral fluid suctioned from the oral cavity during teeth cleaning. In dev our prototypes several versions were considered varying attachment of the brush (V.1 Figure 2) and the size of the fluid evacuation capacity (V.2 and V.3, Figure 2)

## 3. Methods and Materials

### 3.1. Sensory Testing

To determine if the proposed design resulted in a toothbrush that allowed comfortable use. Twenty healthy adult patients (ASA 1) 18–70 years were consented in an IRB approved study. Subjects were conveniently assigned to one of two groups. Subjects in each group received a prototype of the MaxVac toothbrush. Patients were asked to brush their teeth for 2 min and to fill out a survey collecting information concerning ease of use, 10 subjects in each group. Each patient used a newly 3D printed toothbrush head and body.

The timeline began with proof-of-concept snap on vacuum to an existing.

Toothbrush as version 1, followed by all-in-one design and large waste, v. 2 and version 3 a reduction in size and waste reservoir as seen below. Version 2 and versions 3 were used in the experiment for saliva collection and survey measurement with patients.

### 3.2. Microbiological Evaluation Methods

*Streptococcus mutans* strain 25175 was revived from storage in −80 °C on Mitis-Salivarius agar plates. 5 mL of Brain Heart Infusion (BHI) in a 15 mL sterile centrifuge tube was inoculated and incubated overnight under agitation at 37 °C and 250 rpm. The following day 20 mL 1:10 subcultures were made BHI. Initial optical density was recorded, and the tubes were incubated at 37 °C under agitation at 250 RPM until optical density reading between 0.4 and 0.7. Two suction toothbrushes (V.3) were used in the experiment. The toothbrushes were divided into control and experimental groups. 1 mL of exponentially growing *Streptococcus mutans* strain #25175 bacterial culture in Brain Heart Infusion (BHI) medium was suctioned into the internal mechanism of the novel suction toothbrush. For the control brush, three consecutive washes of 10 mL of sterile water were passed through the internal suction mechanism. For the experimental, the washes consisted of 10 mL of sterile water, followed by 10 mL of Listerine Cool Mint, then a final 10 mL sterile water wash. The final washes for both the experimental and control were collected into sterile petri dishes for subsequent serial dilutions and plating. 100 µL of undiluted final washes and 10-fold serial dilutions were plated onto sterile Mitis-Salivarius agar plates. The bacterial subculture used in the experiment was also subjected to 10-fold serial dilutions and 10 µL were plated onto sterile Mitis-Salivarius agar plates to determine starting CFUs/mL and verify viability of cells used in the experiment. Additionally, the remaining collected samples were centrifuged at 3700 rpms for 15 min. The supernatants were removed with laboratory vacuum suction leaving 100 µL of sample to plate. This was then plated onto sterile Mitis-Salivarius agar plates. All plates were left to grow in an incubator at 37 °C for 48 h before CFU/mL counts of each plate were completed.

## 4. Results 

### 4.1. Microbiological Study

Three separate replicates of the experiment resulted with 30, 47, and 18 CFUs counted on the plate containing the 6th serial dilution of the bacterial cell culture indicating an average concentration of bacteria of 3.5 × 10^7^ cells/mL in the original sample used (Table 1). No growth was visible on any plates containing samples from either the experimental or the control after 48 h of incubation. 

### 4.2. Results for the Sensory Testing and Prototype Comparison of Version 2.0 with 3.0

Descriptive Statistics for Saliva and comparison results between toothbrush version 2.0.

Evacuated saliva was collected and measured and versions 2.0 and 3.0 were compared. The median fluid collected was 0.70 mL ± 036 and 0.80 mL ± 0.19 *p* = 0.436 respectively [12]. There were no significant associations found between using two different versions of toothbrushes (Table 2) and the responses of all survey questions (all *p*-value > 0.05). However, for version 3.0 ease of use was best which was the major difference, and 90% in both groups said they would recommend the new toothbrush. 

### 4.3. Conclusions for Both Sensory Testing and Microbiological Testing 

Sensory study: As seen in Table 3, there were no significant associations found between using two different versions of toothbrushes and the responses of all survey questions (all *p*-value > 0.05) pertaining the subjects to toothbrush use. However, importantly, for version 3.0 ease of use was best which was the major difference, and 90% in both groups said they would recommend the new toothbrush.

Conclusions for the micro biological study: CFU/mL growth on plates containing the original cell culture indicate the viability of the bacteria used in the experiment. No growth was visible on plates containing collected following third washes from both the control and the experimental groups. From this, we can conclude that 30 mL of either water or Listerine is effective at reducing the number of bacteria within the internal mechanism to an amount lower than required to cause infection or harm to the user. This experiment indicates that a reasonable disinfection protocol by the average user would prevent infective levels of bacteria from accumulating in the internal mechanism and keep the user safe. Brush heads were also found to be reasonable comfortable; the main body was somewhat heavy in both versions 2 and 3 and finally both groups admitted they would recommend the MaXVac toothbrush (Table 3).

## 5. Discussion

The goal of this project is to develop an assistive technology to facilitate oral hygiene in special needs population while protecting patients from aspiration. In this paper we describe the design and prototype development. We also report on consumer sensory assessment and microbiological determination of consumer cleansing using a common antimicrobial agent. The toothbrush consists of a disposable/replaceable toothbrush head, where oral fluid is drawn in and deposited into a waste compartment located in the brush handle. The waste compartment has a 25 mL capacity. The waste compartment for version 3 is removable and allows oral fluid to be discarded at the end of brushing and cleaning. The sensory testing and comparison of versions 2 and 3 revealed users felt version 3.0 ease of use was best which was the major difference, and 90% in both groups said they would recommend the new toothbrush. The microbiological testing used a common antimicrobial agent used in households, Listerine. Several replicates were done of the experimental and control (water cleaning). This experiment indicates that a reasonable disinfection protocol from the average user would prevent infective/harmful levels of bacteria from accumulating in the internal mechanism and keep the user safe. The lack of bacterial growth on plates after 48 h of incubation in the experiment was considered a success and very exciting news and we look forward to further developing and marketing the new toothbrush. 

Overall, having swallowing difficulties impacts 9.4 million people in the United States [13], and we hope to use this novel toothbrush to close some of the gaps for these patients who need assistance and hopefully offer a solution to reducing aspiration pneumonia. This is is an unmet need when you consider that 406,798 patients were admitted to the hospital in the United States for aspiration pneumonia, from the years, 2002–2012. Of the 406,798 there were 322,598 (79%) were 65 or older. Of these patients 55,477 died and 34,200 had to be placed on a ventilator. Many of these patients as a result likely needed at home care. In the United States there were 1,459,900 home health care patients in 2007 alone and increased from 2000 when there were 1,355,300 home healthcare patients [14,15]. 

Listerine, an antimicrobial mouth rinse, is well known to be effective in killing oral bacteria and readily available, thus we sought to determine if it could disinfect the internal water system of the MaxVac toothbrush. Listerine is reported to completely kill microorganisms within 10 to 30 s; the microbes include but are not limited to methicillin-resistant *Staphylococcus aureus*, *Streptococcus pyogenes*, *Helicobacter pylori*, *Candida albicans*, *Streptococcus mutans*, *Actinomyces viscosus*, *Porphyromonas gingivalis*, *Prevotella intermedia*, and *Aggregatibacter actinomycetemcomitans*. This mouthwash also weakly inactivates human immunodeficiency viruses and COVID-19. Here we demonstrated Listerine was effective in our experimental cleaning set-up after a bacterial broth was suctioned into the toothbrush system. Such a toothbrush and disinfection protocol could help vulnerable populations of patients such as the disabled at risk for aspirating during toothbrushing that could lead to respiratory complications and pneumonia [16].

Many special needs patients can have developmental disabilities such as downs syndrome, cerebral palsy or autism spectrum disorders, these patients usually need diet modification since they may not be able to tolerate solid-food and have swallowing difficulties. They may need thickened liquids or a pureed diet. Soft food will unfortunately also stick to the teeth and contribute to the development of caries and periodontal disease [17,18]. 

The Max Vac toothbrush can be used as an oral care specialty device, offering a solution for young children learning to brush and may ingest too much fluoride which causes mottled enamel. The solution for people with limited capacity and need help with oral care, oral cancer patients, nursing homes, luxury toothbrush for travelers and military defense, as well as space travel since water is self-contained. Electric toothbrushes are an effective and common method for plaque removal. The addition of a vacuum can aid with removal of fluids to prevent pneumonia and other respiratory diseases for both disabled and healthy individuals. 

Current evacuation technologies are bulky and difficult to use outside of dental offices. This optimized toothbrush reduces the risk of infection by preventing harmful bacteria from getting into the lungs. The electric toothbrush would remove plaque and foreign material from the oral cavity while the vacuum would safely evacuate excess saliva, and fluid during brushing. 

The use of the MaxVac in special needs populations will help to prevent excessive plaque buildup. Oral bacteria have also been known to be associated with a variety of systemic diseases. Oral and oropharyngeal microbiota can enter the stomach through swallowed saliva, causing problems with the gut barrier immunity or gut Dysbiosis [19]. These oral bacteria- mediated gut Dysbiosis can result in endotoxemia and systemic inflammation. Future studies will examine the ability of the MaxVac toothbrush to reduce plaque and improve gingivitis levels. Ultimately, we seek to demonstrate that improvement in the oral microbiome could also help improve overall health in the convalescent and special needs population [20,21].

## 6. Patents

Patent application US 16/972,954 and EU 19814148.3 have been filed. A second provisional patent application has been filed in the US 63/402,670.

## Figures and Tables

**Table 1:** Disinfection of toothbrush vacuum lines. *S. mutans* was grown to mid-exponential OD600 growth, CFU/mL was determined at the start of the experiments. The cultures were aspirated through the brush and brushes were disinfected with water or Listerine, and colony counts from the final wash were determined. The data above represents the CFU/mL generated from 3 independent biological replicates.

CFU/mL	Trial 1	Trial 2	Trial 3
Starting culture	3.00 × 10^7^	4.70 × 10^7^	1.82 × 10^7^
Water treatment	0	0	0
Listerine treatment	0	0	0

**Table 2:** Note: Mann-Whitney U test was used for the comparison between saliva volume captured.

Toothbrush Version	N	Mean	Std. Deviation	Median	Mean Rank	*p*-Value
2.0	10	0.70 mL	036	0.70	9.4	*p* = 0.436
3.0	10	0.80 mL	0.19	0.80	11.6	

**Table 3:** Descriptive statistics between the toothbrush version 2.0 and 3.0 the responses of survey question.

Survey Question			Version 2.0		Version 3.0	
		Total	*n*	%	*n*	%
Q 1. What Toothbrush currently	1-manual	8	4	40.0	4	40.0
	2-electric	9	4	40.0	5	50.0
	3-both	3	2	20.0	1	10.0
Q 2. How does the new technology compare	Worse	2	0	0.0	2	20.0
	slightly worse	3	1	10.0	2	20.0
	same	6	2	20.0	4	40.0
	Slightly better	4	3	30.0	1	10.0
	better	5	4	40.0	1	10.0
Q 3. How long do you brush	30 s	0	0	0.0	0	0.0
	1.5 m	12	6	60.0	6	60.0
	2 m	8	4	40.0	4	40.0
Q 4. Ease of use	Too light	0	0	0.0	0	0.0
	light	4	1	10.0	3	30.0
	moderate	7	3	30.0	4	40.0
	Heavy	8	6	60.0	2	20.0
	too heavy	1	0	0.0	1	10.0
Q 5. Would you recommend	Most likely not	1	1	10.0	0	0.0
	likely not	1	0	0.0	1	10.0
	indifferent	0	0	0.0	0	0.0
	Likely yes	10	4	40.0	6	60.0
	most likely yes	8	5	50.0	3	30.0

## Data Availability

Data collected during the panel study or microbiological test are available from Maxine Strickland strickma@sdm.rutgers.edu or Carla Cugini cc1337@sdm.rutgers.edu.
